# Perceptions of Veterinarians and Veterinary Students on What Risk Factors Constitute Medical Disputes and Comparisons between 2014 and 2022

**DOI:** 10.3390/vetsci10030200

**Published:** 2023-03-06

**Authors:** Zih-Fang Chen, Yi-Hsin Elsa Hsu, Jih-Jong Lee, Chung-Hsi Chou

**Affiliations:** 1 Zoonoses Research Center, School of Veterinary Medicine, National Taiwan University, Taipei 10617, Taiwan; 2 Executive Master Program of Business Administration in Biotechnology, Taipei Medical University, Taipei 11031, Taiwan; 3 School of Healthcare Administration, Taipei Medical University, Taipei 11031, Taiwan; 4 Institute of Veterinary Clinical Science, School of Veterinary Medicine, National Taiwan University, Taipei 10617, Taiwan

**Keywords:** Taiwan, questionnaire, veterinarians, veterinary students, cross-section study, medical disputes, factors, complaints, communication education, risk perceptions, Likert scale

## Abstract

**Simple Summary:**

This study investigated the risk factors for medical disputes in the veterinary profession in Taiwan. The research aimed to compare the perceptions of veterinarians and veterinary students and to examine any differences between two surveys conducted in 2014 and 2022. Online validity-tested questionnaires were used to collect data, with 106 (73 veterinarians and 33 students) and 157 (126 veterinarians and 31 students) surveys collected in 2014 and 2022, respectively. The study found that the main causes of medical disputes were poor communication and complaints management, rather than the quality of veterinary care provided. The study also revealed a difference in perceptions between experienced veterinarians and veterinary students, with the latter considering medical skills and clients’ perspectives to be the primary risk factors. However, both groups identified attitudes during interactions and complaint management as key issues. The authors suggest that veterinary education should provide students with more practical experience in medical disputes and complaint management to help bridge the gap in perception between experienced veterinarians and veterinary students. Results from this study have important implications for improving the quality of veterinary care, reducing the risk of medical disputes, and promoting the continuing education of veterinary professionals.

**Abstract:**

This study compared the risk perceptions of medical disputes among veterinarians and veterinary students in Taiwan between 2014 and 2022. Online validity-tested questionnaires were used to collect data, with 106 (73 veterinarians and 33 students) and 157 (126 veterinarians and 31 students) surveys collected in 2014 and 2022, respectively. Respondents would be asked to rate their perceptions on how likely each risk factor constitutes a medical dispute according to their past experiences on a five-point Likert scale from 1 to 5: “Very unlikely, unlikely, neutral, likely, very likely.” The results showed that overall risk perceptions increased significantly in 2022 compared to 2014, with the top risk factors being attitudes during interactions and complaint management among experienced veterinarians. In contrast, students considered medical skills and clients’ perspectives as the top two risk factors, with complaints management ranking as the least significant factor. The findings suggest that effective communication and complaint management are crucial in preventing medical disputes, highlighting the importance of developing these skills in young veterinarians and veterinary students to reduce medical disputes. The study also recommends increasing practical experiences of medical disputes and complaint management in veterinary education to bridge the gap between the perceptions of experienced veterinarians and students.

## 1. Introduction

The number of medical disputes among veterinarians has increased dramatically over the past several decades. As a result, the clients have become increasingly litigious, especially those with companion animals. Compared to economic animals, companion animals are “their families”, [1] not “pets or functional animals” [2] to clients, so the small animal medical disputes pressures are as enormous as human medicine. These medical disputes might cause veterinarians many negative impacts, including mental stress and physical problems [3,4,5,6,7].

Medical disputes include malpractices, such as medical errors, and inadequate communication, such as insufficiently informed and emotional conflicts. Previous research revealed evidence that 80% of claims contained an element of communication breakdown [8] caused by client dissatisfaction [9,10], indicating that communication issues might cause client complaints. The quality of care is not the primary determinant in a client’s decision to initiate a malpractice claim. Instead, most complaints and medical disputes are related to poor communication [8,9,11,12]. Past research outcomes also revealed that emotional conflicts might be caused by disappointing experiences [13,14], dissatisfaction [10,11,12,15], lack of communication [8], and feeling uncared for [4,8,16,17]. These medical disputes may have severe effects on veterinarians. Veterinarians who experienced medical disputes will be more concerned about client complaints and fear litigation, which might cause veterinarians to have intense relationships with future clients, heavier work stress, and even career burnout.

In the past, it was generally believed that communication skills were common sense, but it gradually transformed into one of the core competencies of veterinary professionals [18,19,20,21]. Therefore, the world organization for animal health (WOAH, founded as OIE) announced the core veterinary education curriculum in May 2012, which included “clinical communication courses” to improve communication effectiveness, reduce work pressure and prevent clients’ complaints [16,17,22,23,24,25,26].

Students’ successful transition into clinical practice depends on communication competence by improving client satisfaction and reducing the risk of medical disputes [27]. However, recent veterinary graduates may need more communication skills or abilities from veterinary education or training to manage the inevitable distress in daily work [18]. For example, they try to deliver bad news, talk about euthanasia or terminal disease, show empathetic attitudes, deal with complaints, and even medical expense arguments [28]. Nevertheless, they might learn with “real clients” with colossal stress and gradually become aware of the medical dispute risk and causes [29]. In contrast to the curriculum at school, they have to develop the perceptions of medical disputes via clinical practice and learn the experiences of senior veterinarians as a hidden curriculum [19,30]. Thus, it is essential to understand the differences in perceptions among veterinarians and students to improve communication education and decrease the stress from learning in reality.

Furthermore, this study investigates the perceptions of risk factors that constitute medical disputes and the differences between veterinarians and veterinarians-to-be, the veterinary students. Also, the differences in time are investigated with a comparison of perceptions surveyed in different years. This study primarily focuses on the medical dispute risk factors of companion animals.

## 2. Materials and Methods

### 2.1. Study Design and Questionnaire

The cross-sectional web-based surveys were used to separately investigate the perceptions of risk factors constituting medical disputes among veterinarians and veterinary students in Taiwan in 2014 and 2022. The 2014 survey was financially supported by a project granted by the National Science and Technology Council in Taiwan (NSC 102-2633-S-038-001), and the 2022 survey was completed without government-supporting funds. The questionnaire (which may be found in Supplementary Material) adopted in this study was developed by Hsu (2014) [31]. The questionnaire was developed by literature review and research team discussion and then revised and finalized by focus groups of experts, professors, and veterinarians. The final version questionnaire was valid and reliability tested. The questionnaire included two parts: the first part was the demographic characteristics, including the age, gender, veterinarian or veterinary student, the experience of medical disputes, and the outcomes of medical disputes. The second part was the measures of the perceptions on risk factors constituting medical disputes, including twenty-three risk factors questions belonging to six dimensions: (1) medical skills, (2) modes of communication, (3) the attitude of stakeholders during interactions, (4) medical expenses, (5) complaints management, and 6) clients’ perceptions (see Table 1).

Respondents would be asked to answer their perceptions on how likely each risk factor constitutes medical disputes according to their past experiences on a five-point Likert scale from 1 to 5: “Very unlikely, unlikely, neutral, likely, very likely”. The Likert scale is commonly used to measure the phenomenon in medical science [32]. Past studies have different analyzing approaches to the Likert scale [33,34,35]. The measurement of Likert scales could be analyzed as ordinal or interval data [33,34,35]; some researchers suggested the central tendency could be measured better by using the mean than the median [35,36,37,38] while some suggest vice versa [33,34]. The study was approved by the Research Ethics Committee of National Taiwan University (NTU-REC No.: 202209HS024).

### 2.2. Respondents

This study has surveyed two stakeholders: veterinarians and veterinary students. The questionnaire is an anonymous, self-administered online google form survey. The questionnaire was distributed through the following channels: universities, associations, and social media, such as Facebook and LINE, distributed to the survey participants. The veterinarians who participated were invited by email or social networks, such as clubs of veterinary medical associations and animal hospitals. The participants, veterinary students from five veterinary medicine departments in Taiwan, were invited by email. All participants were asked to answer the online Google questionnaire. The informed consent was shown before replying to the questionnaire, including the survey details, such as the time to complete and confidentiality. They would answer the questionnaire when they accepted the informed consent. The response rate was not reported due to the questionnaire being an anonymous online google form.

### 2.3. Analysis

Demographic characteristics such as age, gender, experiences of medical disputes, and the outcomes of medical disputes in 2014 and 2022 were summarized as numbers, means, and percentages using descriptive statistics. Participants’ perceptions of risk factors in six dimensions were assessed using a five-point Likert scale, and results were analyzed using descriptive statistics (mean and standard deviation) and inferential statistics. The *t*-test was used to compare risk factor perceptions among stakeholders, genders, and experiences of medical disputes in 2014 and 2022. Furthermore, *t*-tests were conducted to compare risk factor perception differences between the two periods for each subgroup (e.g., veterinarians, students, genders, and experiences) separately. All statistical analyses were conducted using SPSS statistical software with a significance level of *p* < 0.05.

## 3. Results

A total of 263 participants completed the survey, with 108 respondents in 2014 and 158 in 2022. However, three participants were excluded from the analysis due to various reasons, such as being less than 20 years old, completing the study in an unrealistic short time (within 1 min), or having too many missing responses. Therefore, the analysis was conducted on 106 respondents in 2014 and 157 in 2022.

### 3.1. Demographic Characteristics

A total of 263 cases responded to the questionnaire, with 106 in 2014 and 157 in 2022. In 2022, there were 126 veterinarians and 31 senior veterinary students, while in 2014, there were 106 veterinarians and 33 senior veterinary students, as shown in Table 2. In 2014, the 20–29 age group and 30–39 age group accounted for 84% of all respondents. In contrast, there were 35 respondents (22%) in the 40–49 age group in 2022, which was five times the percentage in 2014. In 2022, 121 respondents (77%) reported experiencing medical disputes, twice the number in 2014. Simple communication and monetary compensation resolved 75% of medical disputes in 2022, which is about twice the percentage in 2014 (16% and 18%). However, the number of cases resolved by third-party mediation decreased dramatically to one, while the number of cases filed in court increased from one in 2014 to eight in 2022 (5%), a five-fold increase from 2014.

### 3.2. Ranking of the Perceptions of Risk Factors in Six Dimensions

Participants’ perceptions of risk factors in six dimensions were evaluated using a five-point Likert scale, and the results were analyzed using mean and standard deviation. The higher scores indicated that the risk factor was more likely to lead to medical disputes. The six dimensions of risk factors were ranked by the mean score from highest to lowest (see Table 3). When comparing the top risk factors identified by veterinarians in different years, a noteworthy observation emerged. In 2014, respondents identified medical expenses, clients’ perspectives, and attitudes during interactions as the top three risk factors most likely to lead to medical disputes.

In contrast, the top three risk factors in 2022 were attitudes during interactions, complaint management, and medical expenses. Furthermore, the least significant risk factors, or those considered least likely to contribute to medical disputes, were medical skills in 2014 and modes of communication in 2022.

Inexperienced veterinarians and students in 2014 shared similar perceptions of the risk factors most likely to lead to medical disputes. However, in 2022, medical skills were identified as the top risk factor for both groups. When comparing inexperienced veterinarians and students in 2014 and 2022, the top two risk factors identified by students without experience of medical disputes were medical skills and clients’ perspectives, while modes of communication were the least significant risk factor (see Table 4). Interestingly, students ranked complaint management as the fifth risk factor instead of the top first risk ranking among veterinarians with dispute experiences.

### 3.3. Differences in Risk Factor Perception by Demographic Variables in 2014 and 2022

In 2014, 73 veterinarians and 33 students out of the 106 interviewees had significantly different perceptions of the risk of medical disputes caused by medical expenses (*p* < 0.022). For instance, veterinarians gave a risk score of 3.65 ± 1.10 points, ranking it the second-highest level of risk, whereas veterinary students gave 3.07 ± 1.35 points, ranking it fifth. This indicates that veterinarians considered medical expenses to be more likely to lead to medical disputes than the students in 2014.

In 2022, the risk factor with a significant difference in the risk scores given by 126 veterinarians and 31 students was medical skill (*p* < 0.001), with veterinary students giving a score of 4.06 ± 0.53 points, ranking it first, while veterinarians gave 4.14 ± 0.84 points, ranking it fourth. Conversely, regarding the risk of medical skill in 2014, veterinarians ranked it fifth (3.12 ± 1.35), while students ranked it second (3.50 ± 1.36), showing no significant change in the ranking. Nevertheless, there was a difference in risk perception in 2014 that became significantly different in 2022 (see Table 5).

In 2014, there was no difference in risk factor perceptions among males and females across the six dimensions. However, in 2022, women (4.22 ± 0.37) had a significantly higher degree of risk perception for overall risk factors (*p* = 0.005) compared to men (4.04 ± 0.50). Additionally, they had a significantly higher score for the risk of disputes arising from clients’ perspectives (*p* = 0.01) than men (see Table 6). In 2014, respondents with experience (3.83 ± 0.97) believed that the degree of risk caused by medical expenses was significantly higher than that of inexperienced respondents (3.11 ± 1.32) (*p* < 0.002). Consequently, the medical expenses factor ranked first in the group with experience and fifth in the group without experience. Conversely, in 2022, experienced respondents (4.15 ± 0.84) believed that the degree of risk of medical skill factors (*p* = 0.023) was significantly lower than that of inexperienced respondents (4.50 ± 0.64), and the factor ranked fourth, while the no-experience group ranked it first (see Table 7).

### 3.4. Compare Risk Factor Perception Differences between the Two Periods for Each Subgroup

To investigate differences in risk factor perception between the two periods (2014 and 2022) for each subgroup (e.g., males, females, veterinarians, students, experienced and inexperienced in medical disputes), a separate analysis was conducted. All subgroups in 2022 showed significantly higher risk scores than those in 2014 (see Table 8, Table 9 and Table 10). Males in 2014 (*n* = 51) perceived clients’ perspectives and medical expenses as the top two risk factors, while in 2022 (*n* = 66), they considered attitudes during interactions and medical skills as the top two risks. Interestingly, there was no significant difference in risk perception regarding the “clients’ perspective” dimension between the two periods, but the order changed from the first in 2014 to the fifth in 2022. Females had a similar perception of the top two risk factors as males.

In the mean ranking of experienced respondents’ risk perception (see Table 10), those with experience in 2014 (*n* = 53) and inexperienced respondents in 2014 (*n* = 53) both believed that the top two risk factors were medical expenses and clients’ perspectives. In contrast, experienced respondents in 2022 (*n* = 121) perceived attitudes during interactions and complaints management as the top two risks. However, inexperienced respondents (*n* = 36) ranked medical skills as their top risk factor.

In the mean ranking of experienced veterinarians’ risk perception (see Table 11), those with experience in 2014 (*n* = 52) perceived medical expenses and clients’ perspectives as the top two risk factors. In 2022, experienced veterinarians (*n* = 114) perceived attitudes during interactions and complaints management as the top two risks. Inexperienced veterinarians in 2014 (*n* = 21) and students perceived clients’ perspectives and medical skills as the top two risks. In 2022, the top two risk factors perceived by inexperienced veterinarians (*n* = 12) were medical skills and medical expenses, which were similar to students’ perceptions.

## 4. Discussion

This study explores the perceptions of veterinarians and veterinary students in Taiwan on the factors that lead to medical disputes. The study is unique as it compares the perceptions of these two groups using online questionnaires from two different periods. The study’s objectives were to recognize the most significant risk factors, distinguish between the perceptions of veterinarians and veterinary students, and compare the findings of two surveys conducted in 2014 and 2022.

The study’s results revealed that the most significant risk factors for medical disputes among veterinarians and veterinary students were attitudes during interactions, complaint management, and medical expenses. These results differ from the previous survey conducted in 2014, which identified medical expenses, clients’ perspectives, and attitudes during interactions as the top three risk factors for medical disputes. The results suggest that the perception of risk factors has changed over time, and veterinarians and veterinary students have become more aware of the importance of effective communication and complaints management in preventing medical disputes.

Compared to the results obtained from veterinarians in 2014 and 2022; there was a significant increase in overall risk perceptions. This increase in risk perceptions may have been influenced by the continuous veterinary education program implemented since 2016. The Taiwan Veterinarian Act may be one of the reasons for this increase. It requires veterinarians to engage in continuing education and renew their practice license every six years by presenting certificates of completed continuing education to remain eligible to practice last year (2016–2022). In addition, the continuous veterinary education program provides veterinarians with 120 h of training, including 24 h of courses on medical quality improvement, ethics, and laws. Therefore, veterinarians may have been influenced to change their perception of the top two risk factors during interactions with stakeholders and customer complaints management. The increased risk perception may result from the greater emphasis placed on improving the quality of veterinary care and ensuring compliance with ethical and legal standards.

The study found that medical skills and modes of communication were the least significant risk factors that constituted medical disputes. These results suggest that the quality of care provided by veterinarians and veterinary students is not the primary determinant of medical disputes. Instead, poor communication and complaints management were the leading causes of medical disputes, similar to previous studies [11,39]. The findings highlight the importance of improving the communication skills of veterinarians and veterinary students, developing effective complaint management strategies, and reducing the risk of medical disputes. On the other hand, the modes of communication mean the process of informed consent, such as explanations and information. Effective modes of communication might have become the standard operation procedure (SOP) and online-friendly tools to help clients get information during these years, as previously reported [26,40].

The study’s results also revealed significant differences in the perception of risk factors between veterinarians and veterinary students. For instance, in 2014, veterinarians considered medical expenses (*p* = 0.022) more likely to lead to medical disputes than veterinary students [28]. However, in 2022, veterinary students ranked medical skills (*p* < 0.001) as the top risk factor, while veterinarians ranked it fourth. Moreover, the study found that experienced veterinarians had a different perception of risk factors than inexperienced veterinarians and veterinary students. Experienced veterinarians perceived attitudes during interactions and complaint management as the top risk factors for medical disputes. In contrast, inexperienced veterinarians and veterinary students ranked clients’ perspectives and medical skills as the top risk factors. These results suggest that the perception of risk factors may change as veterinarians gain more experience and become more aware of the importance of effective communication and complaint management. Although veterinary students’ successful transition into clinical practice depends on communication competence, their perceptions seem significantly different from senior veterinarians; it was similar to previous studies [27,41]. Thus, increased employer cooperation in regard to continued education and research into best practices for tertiary institution–employer collaboration needs to be developed.

The study is not without limitations. One limitation is that it was conducted in Taiwan, and the population of veterinarians and veterinary students is relatively small compared to other countries. Therefore, the results may not be generalizable to other countries with different healthcare systems and cultural backgrounds. Another limitation is that the study used a cross-sectional survey design, which cannot establish causality. As a result, the causal relationships between the variables cannot be determined. A third limitation is that the study relied on self-reported data, which may be subject to social desirability bias. Participants may have provided answers that they believed were socially desirable rather than reflecting their true perceptions. Fourth, the study did not investigate the factors contributing to the change in perception of risk factors over time. Understanding these factors could provide valuable insights into the reasons for the changes in perception. Lastly, the Likert scale is commonly used as interval data in the medical area, although it is considered ordinal data. Therefore, the data were analyzed as a median rather than a mean.

Despite these limitations, the study provides valuable insights into the perception of risk factors that cause medical disputes among veterinarians and veterinary students. Future research can explore the factors contributing to changes in risk perception over time, compare the perception of risk factors in different countries, and examine the effectiveness of communication strategies and complaint management skills in reducing the risk of medical disputes. Additionally, further research could investigate the efficacy of different educational programs in developing effective communication and complaint management skills in young veterinarians and veterinary students. Understanding these factors can help improve the quality of care for companion animals, reduce the risk of medical disputes, and enhance the competency of veterinary professionals.

In conclusion, the study provides valuable insights into the perception of risk factors that cause medical disputes among veterinarians and veterinary students. The results suggest effective communication and complaint management are crucial in preventing medical disputes. The study highlights the importance of developing effective communication strategies and complaint management skills for young veterinarians and veterinary students, reducing the risk of medical disputes, and improving the quality of care for companion animals. Veterinary education might be suggested to increase the practical experience of medical disputes and complaint management to help students overcome the gaps and gain the competency to transition into clinical practice successfully.

## Figures and Tables

**Table 1:** Six dimensions of the questionnaire for measures of perceptions towards medical disputes.

Dimensions	Content
1. Medical skills	Misdiagnosis leading to worsening of patient condition. Inappropriate/improper hospital care or treatment procedures.
2. Modes of communication	Explanations from the veterinarians to the clients are too simple. Use of too many medical terms during explanation with no clear supplementary explanation. The clients have doubts about the treatment but fail to enquire or get an unsatisfactory answer from the veterinarians before the treatment. A gap between clients’ expected and actual treatment outcomes due to a lack of information for decision-making before the treatment. No precise decision being made by the clients’ families during the discussion of subsequent treatment procedures with the veterinarian. The veterinarians do not provide supplementary paper documents to the clients to raise their knowledge level about the treatment.
3. The attitude of stakeholders during interactions	The veterinarians try to make the clients reluctantly accept the recommended diagnosis and treatments. The veterinarians do not respond appropriately or take appropriate steps to solve the clients’ concerns. The veterinarians do not give feelings of support and encouragement during interactions, resulting in the clients’ thinking that the veterinarian may not be concerned about the health of sick animals. When the clients’ and veterinarians’ views on the animals’ condition differ, the client tends to stick with their subjective perception.
4. Medical expenses	The veterinarians do not explain to the clients in advance the possible total medical expenses. The veterinarians do not clearly explain the possible medical costs for each treatment procedure. The disparity between clients’ expected and actual medical costs.
5. Complaints management	The veterinarians did nothing to respond to the complaints in time. Absence of senior staff to attend to the complaints. The complaint resolution process was done with inappropriate attitudes and improper ways. Staff members do not have relevant professional training to handle the complaints.
6. Clients’ perceptions	The clients depend on self-gathered information. The clients have many questions about treatment and lack trust in the interaction. The clients’ misconception of pets being taken care of by the veterinarian. The clients may have the motives of extortion.

**Table 2:** Demographic characteristics distribution of participants in 2014 and 2022.

Characteristics	2014 (*n* = 106)		2022 (*n* = 157)	
	*n*	%	*n*	%
Stakeholder’s position				
Veterinarian	73	68.90%	126	80.30%
Veterinary student	33	31.10%	31	19.70%
Age				
20–29 years old	53	50.00%	58	36.90%
30–39 years old	36	34.00%	56	35.70%
40–49 years old	5	4.70%	35	22.30%
50+ years old	12	11.30%	8	5.10%
Gender				
Male	51	48.10%	66	42.00%
Female	55	51.90%	91	58.00%
Experiences of medical disputes				
Experienced	53	50.00%	121	77.10%
Inexperienced	53	50.00%	36	22.90%
Outcomes of medical disputes				
No idea	54	50.90%	12	7.60%
Resolved by simple communication	17	16.00%	56	35.70%
Resolved by communication and reconciliation via money compensation or medical fee reduction	19	17.90%	63	40.10%
Resolved by simple third-party involvement/mediation	7	6.60%	1	0.60%
Resolved by third party involvement and reconciliation via money compensation or medical fee reduction	8	7.50%	17	10.80%
Unresolved even with third party involvement and the complaint was filed in the court	1	0.90%	8	5.10%

**Table 3:** Ranking of the perceptions of risk factors in six dimensions for veterinarians and students in 2014 and 2022.

Risk Factors Dimensions	Veterinarians				Students			
	2014 (*n* = 73)		2022 (*n* = 126)		2014 (*n* = 33)		2022 (*n* = 31)	
	Mean	Rank ^1^	Mean	Rank	Mean	Rank	Mean	Rank
Clients’ perspectives	3.70	1	4.07	5	3.53	1	4.17	2
Medical expenses	3.65	2	4.17	3	3.07	5	4.14	3
Attitudes during interactions	3.45	3	4.26	1	3.22	4	4.14	3
Modes of communication	3.20	4	3.98	6	2.99	6	3.84	6
Medical skills	3.12	5	4.14	4	3.50	2	4.61	1
Complaints management	3.09	6	4.20	2	3.31	3	4.07	5

^1^ Rank by the mean from highest to lowest.

**Table 4:** Ranking of the perceptions of risk factors in six dimensions for inexperienced Veterinarians and students in 2014 and 2022.

Risk Factors Dimensions	Students without Experience				Veterinarians without Experience			
	2014 *n* = 32		2022 *n* = 24		2014 *n* = 21		2022 *n* = 12	
	Mean	Rank ^1^	Mean	Rank	Mean	Rank	Mean	Rank
Clients’ perspectives	3.48	1	4.18	3	3.58	1	4.17	5
Medical skills	3.45	2	4.60	1	3.45	2	4.29	1
Complaints management	3.27	3	4.02	5	3.20	5	4.25	3
Attitudes during interactions	3.16	4	4.23	2	3.33	3	4.23	4
Medical expenses	3.01	5	4.14	4	3.25	4	4.28	2
Modes of communication	2.96	6	3.80	6	3.17	6	4.01	6

^1^ Rank by the mean from highest to lowest.

**Table 5:** The risk factor perception differences between veterinarians and students in 2014 and 2022.

Risk Factors Dimensions	2014					2022				
	Stakeholder’s Position					Stakeholder’s Position				
	Veterinarians *n* = 73		Students *n* = 33			Veterinarians *n* = 126		Students *n* = 31		
	Mean	SD	Mean	SD	*p*	Mean	SD	Mean	SD	*p*
Risk_all ^1^	3.37	0.80	3.27	0.97	0.599	4.14	0.40	4.16	0.56	0.769
client_p	3.70	1.05	3.53	1.38	0.492	4.07	0.65	4.17	0.59	0.417
inter_att	3.45	1.00	3.22	1.25	0.324	4.26	0.57	4.14	0.78	0.324
med_exp	3.65	1.10	3.07	1.35	0.022 *	4.17	0.67	4.14	0.89	0.823
med_sk	3.12	1.35	3.50	1.36	0.179	4.14	0.84	4.61	0.53	<0.001 ***
complaint	3.09	1.10	3.31	1.33	0.364	4.20	0.60	4.07	0.75	0.309
commu	3.20	0.92	2.99	0.91	0.279	3.98	0.56	3.84	0.73	0.251

^1^ Risk_all: Average scores of all the risk factors including medical skills (med_sk), modes of communication (commu), attitudes of stakeholders during the interaction (inter_att), medical expenses (med_exp), complaints management (complaint), and clients’ perceptions (client_p). *p* value < 0.05 *; *p* value < 0.001 ***.

**Table 6:** The risk factor perception differences by gender in 2014 and 2022.

Risk Factors Dimensions	2014					2022				
	Gender					Gender				
	Male *n* = 51		Female *n* = 55			Male *n* = 66		Female *n* = 91		
	Mean	SD	Mean	SD	*p*	Mean	SD	Mean	SD	*p*
Risk_all ^1^	3.43	0.76	3.25	0.93	0.302	4.04	0.50	4.22	0.37	0.017 *
client_p	3.71	0.87	3.59	1.38	0.607	3.93	0.71	4.20	0.56	0.01 **
inter_att	3.49	0.99	3.27	1.16	0.295	4.14	0.72	4.30	0.53	0.133
med_exp	3.62	1.01	3.33	1.36	0.212	4.06	0.79	4.25	0.65	0.101
med_sk	3.29	1.19	3.18	1.50	0.67	4.13	0.88	4.31	0.75	0.173
complaint	3.24	1.11	3.08	1.24	0.478	4.11	0.63	4.22	0.64	0.289
commu	3.21	0.94	3.08	0.89	0.466	3.86	0.62	4.01	0.57	0.105

^1^ Risk_all: Average scores of all the risk factors including medical skills (med_sk), modes of communication (commu), attitudes of stakeholders during the interaction (inter_att), medical expenses (med_exp), complaints management (complaint), and clients’ perceptions (client_p). *p* value < 0.05 *; *p* value < 0.01 **.

**Table 7:** The risk factor perception differences in experiences of medical disputes in 2014 and 2022.

Risk Factors Dimensions	2014					2022				
	Experiences of Medical Disputes					Experiences of Medical Disputes				
	Experienced *n* = 53		Inexperienced *n* = 53			Experienced *n* = 121		Inexperienced *n* = 36		
	Mean	SD	Mean	SD	*p*	Mean	SD	Mean	SD	*p*
Risk_all ^1^	3.41	0.79	3.27	0.92	0.411	4.13	0.40	4.18	0.55	0.587
client_p	3.77	1.04	3.52	1.27	0.279	4.06	0.62	4.17	0.69	0.348
inter_att	3.52	0.94	3.23	1.20	0.173	4.24	0.59	4.23	0.72	0.943
med_exp	3.83	0.97	3.11	1.32	0.002 **	4.16	0.68	4.19	0.82	0.852
med_sk	3.02	1.37	3.45	1.32	0.1	4.15	0.84	4.50	0.64	0.023 *
complaint	3.07	1.09	3.25	1.26	0.435	4.20	0.59	4.10	0.77	0.393
commu	3.23	0.95	3.05	0.88	0.306	3.97	0.55	3.87	0.73	0.368

^1^ Risk_all: Average scores of all the risk factors including medical skills (med_sk), modes of communication (commu), attitudes of stakeholders during the interaction (inter_att), medical expenses (med_exp), complaints management (complaint), and clients’ perceptions (client_p). *p* value < 0.05 *; *p* value < 0.01 **.

**Table 8:** Comparisons of the risk factor perceptions differences between 2014 and 2022 for gender subgroups.

Risk Factors Dimensions	Gender									
	Male *n* = 117					Female *n* = 146				
	2014 *n* = 51		2022 *n* = 66			2014 *n* = 55		2022 *n* = 91		
	Mean	SD	Mean	SD	*p*	Mean	SD	Mean	SD	*p*
Risk_all ^1^	3.43	0.76	4.04	0.50	<0.001 ***	3.25	0.93	4.22	0.37	<0.001 ***
client_p	3.71	0.87	3.93	0.71	0.131	3.59	1.38	4.20	0.56	0.003 **
inter_att	3.49	0.99	4.14	0.72	<0.001 ***	3.27	1.16	4.30	0.53	<0.001 ***
med_exp	3.62	1.01	4.06	0.79	0.013 **	3.33	1.36	4.25	0.65	<0.001 ***
med_sk	3.29	1.19	4.13	0.88	<0.001 ***	3.18	1.50	4.31	0.75	<0.001 ***
complaint	3.24	1.11	4.11	0.63	<0.001 ***	3.08	1.24	4.22	0.64	<0.001 ***
commu	3.21	0.94	3.86	0.62	<0.001 ***	3.08	0.89	4.01	0.57	<0.001 ***

^1^ Risk_all: Average scores of all the risk factors including medical skills (med_sk), modes of communication (commu), attitudes of stakeholders during the interaction (inter_att), medical expenses (med_exp), complaints management (complaint), and clients’ perceptions (client_p). *p* value < 0.01 **; *p* value < 0.001 ***.

**Table 9:** Comparisons of the risk factor perceptions differences between 2014 and 2022 for veterinarians and students subgroup.

Risk Factors Dimensions	Stakeholder’s Position									
	Veterinarians					Students				
	2014 *n* = 73		2022 *n* = 126			2014 *n* = 33		2022 *n* = 31		
	Mean	SD	Mean	SD	*p*	Mean	SD	Mean	SD	*p*
Risk_all ^1^	3.37	0.80	4.14	0.40	<0.001 ***	3.27	0.97	4.16	0.56	<0.001 ***
client_p	3.70	1.05	4.07	0.65	0.008 **	3.53	1.38	4.17	0.59	0.019 *
inter_att	3.45	1.00	4.26	0.57	<0.001 ***	3.22	1.25	4.14	0.78	<0.001 ***
med_exp	3.65	1.10	4.17	0.67	<0.001 ***	3.07	1.35	4.14	0.89	<0.001 ***
med_sk	3.12	1.35	4.14	0.84	<0.001 ***	3.50	1.36	4.61	0.53	<0.001 ***
complaint	3.09	1.10	4.20	0.60	<0.001 ***	3.31	1.33	4.07	0.75	0.006 **
commu	3.20	0.92	3.98	0.56	<0.001 ***	2.99	0.91	3.84	0.73	<0.001 ***

^1^ Risk_all: Average scores of all the risk factors including medical skills (med_sk), modes of communication (commu), attitudes of stakeholders during the interaction (inter_att), medical expenses (med_exp), complaints management (complaint), and clients’ perceptions (client_p). *p* value < 0.05 *; *p* value < 0.01 **; *p* value < 0.001 ***.

**Table 10:** Comparisons of the risk factor perceptions differences between 2014 and 2022 for medical disputes experience subgroup.

Risk Factors Dimensions	Experiences of Medical Disputes									
	Experienced					Inexperienced				
	2014 *n* = 53		2022 *n* = 121			2014 *n* = 53		2022 *n* = 36		
	Mean	SD	Mean	SD	*p*	Mean	SD	Mean	SD	*p*
Risk_all ^1^	3.41	0.79	4.13	0.40	<0.001 ***	3.27	0.92	4.18	0.55	<0.001 ***
client_p	3.77	1.04	4.06	0.62	0.061	3.52	1.27	4.17	0.69	0.003 **
inter_att	3.52	0.94	4.24	0.59	<0.001 ***	3.23	1.20	4.23	0.72	<0.001 ***
med_exp	3.83	0.97	4.16	0.68	0.028 *	3.11	1.32	4.19	0.82	<0.001 ***
med_sk	3.02	1.37	4.15	0.84	<0.001 ***	3.45	1.32	4.50	0.64	<0.001 ***
complaint	3.07	1.09	4.20	0.59	<0.001 ***	3.25	1.26	4.10	0.77	<0.001 ***
commu	3.23	0.95	3.97	0.55	<0.001 ***	3.05	0.88	3.87	0.73	<0.001 ***

^1^ Risk_all: Average scores of all the risk factors including medical skills (med_sk), modes of communication (commu), attitudes of stakeholders during the interaction (inter_att), medical expenses (med_exp), complaints management (complaint), and clients’ perceptions (client_p). *p* value < 0.05 *; *p* value < 0.01 **; *p* value < 0.001 ***.

**Table 11:** Comparisons of the risk factor perceptions differences between 2014 and 2022 for veterinarians with/without experiences subgroup.

Risk Factors Dimensions	Veterinarians with Experiences					Veterinarians without Experiences				
	2014 *n* = 52		2022 *n* = 114			2014 *n* = 21		2022 *n* = 12		
	Mean	SD	Mean	SD	*p*	Mean	SD	Mean	SD	*p*
Risk_all ^1^	3.38	0.77	4.13	0.41	<0.001 ***	3.33	0.90	4.20	0.37	0.003 **
client_p	3.75	1.03	4.05	0.64	0.05 *	3.58	1.12	4.17	0.76	0.12
inter_att	3.49	0.93	4.26	0.59	<0.001 ***	3.33	1.18	4.23	0.36	0.003 **
med_exp	3.81	0.96	4.16	0.68	0.02 *	3.25	1.32	4.28	0.62	0.005 **
med_sk	2.98	1.36	4.12	0.85	<0.001 ***	3.45	1.30	4.29	0.75	0.05
complaint	3.04	1.08	4.20	0.60	<0.001 ***	3.20	1.18	4.25	0.68	0.003 **
commu	3.21	0.95	3.97	0.56	<0.001 ***	3.17	0.85	4.01	0.60	0.005 **

^1^ Risk_all: Average scores of all the risk factors including medical skills (med_sk), modes of communication (commu), attitudes of stakeholders during the interaction (inter_att), medical expenses (med_exp), complaints management (complaint), and clients’ perceptions (client_p). *p* value < 0.05 *; *p* value < 0.01 **; *p* value < 0.001 ***.

## Data Availability

Data sharing not applicable.

## Supplementary Materials

The following are available online at https://www.mdpi.com/article/10.3390/vetsci10030200/s1, Supplementary Material: Questionnaire.
