# Supplementary material for: Perceptions of Veterinarians and Veterinary Students on What Risk Factors Constitute Medical Disputes and Comparisons between 2014 and 2022
Source: Vet Sci. 2023 Mar 6;10(3):200. doi: 10.3390/vetsci10030200 (PMC10056102; doi:10.3390/vetsci10030200)
Supplement: Supplementary file 1 [file vetsci-10-00200-s001.zip › vetsci-2179180-supplementary.pdf]

I. Interviewee's information

Birthdate (this research will only include participants of 21 years and above)

1. Gender: □ Male □ Female
2. Stakeholder's position:

□ a) Veterinarian
□ b) Veterinary Student.
□ c) Client.

II. Survey on experience of companion animal medical disputes.

(Animal medical disputes: Disputes related to treatment of companion animals)

1. Have you ever had an experience of animal medical disputes?
□ a) Yes (includes the complainant, respondent and a person who has knowledge of the complaint) (Please go to the next question)
□ b) No (Please go to question 3)
2. Following the previous question, how was the complaint resolved?
□ a) Resolved by simple communication.
□ b) Resolved by communication and reconciliation via money compensation or medical fee reduction.
□ c) Resolved by simple third-party involvement/mediation.
□ d) Resolved by third-party involvement and reconciliation via money compensation or medical fee reduction.
□ e) Unresolved even with third-party involvement and the complaint was filed in the court.
3. Which of the following reasons do you think can likely lead to animal medical related disputes?

| Degree to which a cause may lead to a complaint | | |
|---|---|---|
| 1 - Very unlikely | 2 - Unlikely | 3 - Neutral |
| 4 - Likely | 5 - Very likely | |

| Degree to which a cause will lead to a complaint | | | | | | Possible causes leading to animal medical related disputes or customer complaints |
|---|---|---|---|---|---|---|
| | 1 | 2 | 3 | 4 | 5 | Please tick [✓] the most appropriate answer for you in the left column. |
| (1) Medical skill (Note: "Veterinarian" means chief attending veterinarian of Veterinary Hospital; It also includes attending veterinarian and other hospital staff; "Client" means pet owner) | | | | | | |
| | | | | | | 1.1 Misdiagnosis leading to worsening of patient condition. |
| | | | | | | 1.2 Inappropriate/improper hospital care or treatment procedures. |
| (2) Modes of communication | | | | | | |
| | | | | | | 2.1 Explanations from the veterinarians to the clients are too simple. |
| | | | | | | 2.2 Use of too many medical terms during explanation with no clear supplementary explanation. |
| | | | | | | 2.3 The clients have doubts about the treatment but fail to enquire or get an unsatisfactory answer from the veterinarians before the treatment. |

| | | | | | | |
|---|---|---|---|---|---|---|
| | | | | | | 2.4 A gap between clients expected and actual treatment outcomes due to a lack of information for decision-making before the treatment. |
| | | | | | | 2.5 No precise decision being made by the clients' families during the discussion of subsequent treatment procedures with the veterinarian. |
| | | | | | | 2.6 The veterinarians do not provide supplementary paper documents to the clients to raise their knowledge level about the treatment. |
| (3) Attitudes during interactions | | | | | | |
| | | | | | | 3.1 The veterinarians try to make the clients reluctantly accept the recommended diagnosis and treatments. |
| | | | | | | 3.2 The veterinarians do not respond appropriately or take appropriate steps to solve the clients' concerns. |
| | | | | | | 3.3 The veterinarians do not give feelings of support and encouragement during interactions, resulting in the clients' thinking that the veterinarian may not be concerned about the health of sick animals. |
| | | | | | | 3.4 When the clients' and veterinarians' views on the animals' condition differ, the client tends to stick with their subjective perception. |
| (4) Medical expenses | | | | | | |
| | | | | | | 4.1 The veterinarians do not explain to the clients in advance the possible total medical expenses. |
| | | | | | | 4.2 The veterinarians do not clearly explain the possible medical costs for each treatment procedure. |
| | | | | | | 4.3 The disparity between clients expected and actual medical costs. |
| (5) Complaints management | | | | | | |
| | | | | | | 5.1 The veterinarians did nothing to respond to the complaints in time. |
| | | | | | | 5.2 Absence of senior staff to attend to the complaints. |
| | | | | | | 5.3 The complaint resolution process was done with inappropriate attitudes and improper ways. |
| | | | | | | 5.4 Staff members do not have relevant professional training to handle the complaints. |
| (6) Clients' perceptions | | | | | | |
| | | | | | | 6.1 The clients depend on self-gathered information. |
| | | | | | | 6.2 The clients have many questions about treatment and lack trust in the interaction. |
| | | | | | | 6.3 The clients' misconception of pets being taken care of by the veterinarian. |
| | | | | | | 6.4 The clients may have the motives of extortion. |
| Other causes (except the before mentioned causes), please explain: | | | | | | |
